# Heparan Sulfates Regulate Axonal Excitability and Context Generalization through Ca^2+^/Calmodulin-Dependent Protein Kinase II

**DOI:** 10.3390/cells12050744

**Published:** 2023-02-25

**Authors:** Inseon Song, Tatiana Kuznetsova, David Baidoe-Ansah, Hadi Mirzapourdelavar, Oleg Senkov, Hussam Hayani, Andrey Mironov, Rahul Kaushik, Michael Druzin, Staffan Johansson, Alexander Dityatev

**Affiliations:** 1 Molecular Neuroplasticity Group, German Center for Neurodegenerative Diseases (DZNE), 39120 Magdeburg, Germany; 2 Department of Integrative Medical Biology, Umeå University, 90187 Umeå, Sweden; 3 Medizinische Fakultät, Otto-von-Güricke-Universität Magdeburg, 39120 Magdeburg, Germany; 4 Center for Behavioral Brain Sciences (CBBS), 39106 Magdeburg, Germany

**Keywords:** extracellular matrix, synaptic plasticity, context discrimination, axon initial segment, axonal excitability, ankyrin G

## Abstract

Our previous studies demonstrated that enzymatic removal of highly sulfated heparan sulfates with heparinase 1 impaired axonal excitability and reduced expression of ankyrin G at the axon initial segments in the CA1 region of the hippocampus ex vivo, impaired context discrimination in vivo, and increased Ca^2+^/calmodulin-dependent protein kinase II (CaMKII) activity in vitro. Here, we show that in vivo delivery of heparinase 1 in the CA1 region of the hippocampus elevated autophosphorylation of CaMKII 24 h after injection in mice. Patch clamp recording in CA1 neurons revealed no significant heparinase effects on the amplitude or frequency of miniature excitatory and inhibitory postsynaptic currents, while the threshold for action potential generation was increased and fewer spikes were generated in response to current injection. Delivery of heparinase on the next day after contextual fear conditioning induced context overgeneralization 24 h after injection. Co-administration of heparinase with the CaMKII inhibitor (autocamtide-2-related inhibitory peptide) rescued neuronal excitability and expression of ankyrin G at the axon initial segment. It also restored context discrimination, suggesting the key role of CaMKII in neuronal signaling downstream of heparan sulfate proteoglycans and highlighting a link between impaired CA1 pyramidal cell excitability and context generalization during recall of contextual memories.

## 1. Introduction

Heparan sulfate proteoglycans (HSPGs) harbor long chains of variously sulfated polysaccharide residues. There are membrane-bound HSPGs, such as syndecans and glypicans, and secreted HSPGs, including agrin, perlecan, and collagen type XVIII. An increasing number of studies demonstrate that HSPGs have an important role in the nervous system during development and adulthood.

In the mouse brain, syndecan-1 and glypican-4 are highly expressed in the neural tube, where the precursor cells are proliferating [1]. These HSPGs are important for the proliferation of neural precursor cells and play a role as synaptic organizing molecules during synaptogenesis. Their heparan sulfate (HS) chains are essential for this role. Glypican 4 is bound to the presynaptic membrane via a GPI anchor and interacts with the postsynaptic protein, LRRTM4 (leucine-rich repeat transmembrane neuronal proteins), forming a trans-synaptic complex. This complex recruits other synaptic molecules to the synaptic cleft, contributing to the maturation of excitatory synapses. Mice deficient in glypican 4 exhibit a decreased number of synapses along with decreased expression of postsynaptic glutamate receptor subunit GluA1 and increased retention of presynaptic neuronal pentraxin 1 [2].

Syndecans are differentially expressed in various neural cell types and exhibit differential subcellular localization in neurons [3]. Contrary to glypicans lacking a cytoplasmic domain, transmembrane syndecans interact with specific cytoplasmic binding partners, such as Ca^2+^/calmodulin-dependent serine protein kinase (CASK), syntenin, synectin, and synbindin [4,5,6,7]. Syndecan 2 is highly expressed in synapses and influences activities of postsynaptic scaffolding proteins, thereby contributing to filopodia and dendritic spine formation [8]. Overexpression of full-length syndecan 2 in cultured immature hippocampal neurons accelerates dendritic spine formation, while a syndecan 2 deletion mutant that lacks the ability to bind to synthenin and CASK does not support spine maturation [4,9]. The association of cortactin and fyn with syndecan is increased rapidly after induction of long-term potentiation (LTP), while inclusion of soluble syndecan 3 into the rat hippocampal slices inhibits high-frequency stimulation-induced LTP [10]. Furthermore, syndecan 3 knockout mice exhibit strongly enhanced LTP and impaired hippocampus-dependent memory [11]. Secreted HSPG agrin is also involved in filopodia and dendritic spine formation. While downregulation of agrin in the cultured neurons in vitro and in vivo reduces the number of dendritic filopodia, overexpression of agrin in rodent hippocampal neurons stimulates filopodia formation in vitro [12].

An increasing number of structural, pharmacological, and genetic studies suggest a key role of the HS chains carried by HSPGs in mediating their activities. Interestingly, HSs bind to receptor protein tyrosine phosphatase sigma (RPTPσ) at the same site as chondroitin sulfates. Crystallographic analyses of this site reveal conformational plasticity that can accommodate diverse glycosaminoglycans with comparable affinities. HSs induced RPTPσ ectodomain oligomerization, stimulating neurite outgrowth. The oligomerization was inhibited by chondroitin sulfates, resulting in impaired neurite outgrowth [13]. In acute hippocampal slices, treatment with a mixture of heparinases 1 and 3, which removes highly and low sulfated HSs, respectively, impaired LTP of synaptic transmission [10,14]. This treatment also prevented the increase in the number of spines after induction of N-methyl-D-aspartate (NMDA) receptor-dependent LTP [14]. Conditional ablation of Ext1, a gene involved in HS synthesis, in a subpopulation of pyramidal neurons leads to an autistic phenotype [15], providing genetic evidence for the importance of HSs in shaping brain function on many levels, from cellular properties to complex behaviors. More targeted ablation of HSs on neurexin-1 also revealed structural and functional deficits at central synapses. HS directly binds postsynaptic partners neuroligins and LRRTMs [16].

Considering the high heterogeneity of HSs, we focused on a highly sulfated subset of HSs (HSHSs), which could be digested by heparinase 1. Such treatment of cultured hippocampal neurons resulted in a reduction in the mean firing rate of neurons [17,18], despite the upregulation of GluA1 protein expression [17]. Acute treatment of hippocampal slices with heparinase 1 reduced CA1 pyramidal cellular excitability and impaired hippocampal LTP [19]. Altered expression of ankyrin G (AnkG), as one of the major organizing proteins at the axon initial segment (AIS) in heparinase 1-treated hippocampal slices, led us to the hypothesis that HSHSs are involved in the modulation of neuronal activity through the changes in the AIS composition and function. Injection of heparinase 1 before fear conditioning impaired context discrimination [19], validating the importance of HSHSs at the systemic level.

Based on previous in vitro findings of increased autophosphorylation levels of CaMKII α and β isoforms after heparinase 1 treatment [17], we hypothesized that CaMKII is the key molecule involved in the modulation of axonal excitability due to a loss of HSHSs and provided in vitro and in vivo evidence verifying this hypothesis biochemically, immunocytochemically, and electrophysiologically. Our studies show that an increased level of autophosphorylated CaMKII in heparinase-treated neurons is responsible for reduced neuronal excitability, altered expression of AnkG in the AIS of CA1 pyramidal neurons, and impaired contextual discrimination.

## 2. Materials and Methods

### 2.1. Immunoblot Analysis

To access the level of endogenous CaMKII isoform expression and the level of its phosphorylation, murine hippocampal slices (treated with intact or heat-inactivated heparinase 1 in the same way as for electrophysiological recordings) were snap-frozen in isopropanol pre-cooled on dry ice. Later samples were homogenized in radioimmunoprecipitation assay (RIPA) buffer (ThermoFisher Scientific, Rockford, IL, USA) containing a protease inhibitor cocktail (Sigma-Aldrich P1860, St. Louis, MO, USA), a serine/threonine phosphatase inhibitor (Sigma-Aldrich P0044, St. Louis, MO, USA), and a tyrosine phosphatase inhibitor (Sigma-Aldrich P5726, St. Louis, MO, USA) using a glass tissue homogenizer. Non-soluble proteins were separated via centrifugation at 20,000 g for 15 min at 4 °C. The protein concentration of individual samples was measured using a DC Protein Assay (Bio-Rad, Hercules, CA, USA). A total of 10–30 μg of extract was resuspended in reducing (5.0% 2-mercaptoethanol) sample buffer (Bio-Rad, Hercules, CA, USA) and boiled at 100 °C for 5 min, separated via SDS-PAGE on 10% acrylamide gels, and transferred to the polyvinylidene difluoride membranes. Membranes were blocked for 1 h at room temperature with 5% Blotting-Grade Blocker (Bio-Rad, 1706404, Hercules, CA, USA) in Tris-buffered saline with Tween20 (TBS-T buffer), probed with appropriate primary antibody at 4 °C overnight and then for 1 h at room temperature with horseradish peroxidase (HRP)-conjugated secondary antibodies.

To estimate the total expression of α and β forms of CaMKII, mouse anti-CaMKII (G1, sc-5306; 1:200–1:1000) from Santa-Cruz (Paso Robles, CA, USA) was used. To induce activation of CaMKII, rabbit anti-phospho Thr 286/287 CaMKII (p1005–286; 1:1000) from PhosphoSolutions (Aurora, CO, USA) was applied. To evaluate the glyceraldehyde-3-phosphate dehydrogenase (GAPDH) level, mouse anti-GAPDH (MAB374; 1:15.000–20.000) from Millipore (Bedford, MA, USA) was used. HRP-conjugated secondary antibodies were donkey anti-rabbit (NA934V) from GE Healthcare (Buckinghamshire, UK), or goat anti-mouse (115-035-146) from Jackson ImmunoResearch (West Grove, PA, USA). Acquisition of chemiluminescent signal and densitometric analysis were performed using an Odyssey Fc Imaging System (LI-COR, NE, USA) and Image Studio Lite 5.2.5 software, respectively. The total levels of α or β forms of CaMKII and phospho Thr 286/287 levels were standardized to the level of loading control (GAPDH) in each sample. Standardized values were further normalized to the randomly chosen control sample (loaded in each gel). To evaluate CaMKII phosphorylation on Thr 286/287, the ratios of phospho-Thr 286/287 signal to the total amount of CaMKII protein were calculated.

For statistical evaluation and the graphical representation of the data, the OriginPro 2022 9.9.0.225 software was used. The average ± SEM (standard error of mean) was calculated for control and experimental (heparinase-treated) groups, normalized to randomly chosen control samples. Statistical evaluation was carried out using a Mann–Whitney–Wilcoxon test.

### 2.2. Slice Preparation and In Vitro Electrophysiology

Acute hippocampal slices were prepared as described elsewhere [19] from 4- to 5-week-old male C57Bl/6 mice 1 day after injection with Heparinase 1, Ctrl (heat-inactivated Heparinase 1), or Heparinase 1 + autocamtide-2-related inhibitory peptide (AIP) into hippocampal CA1 area, which described below (Section 2.4). Transverse 350 µm thick hippocampal slices were obtained in ice-cold slice solution containing (in mM) 240 sucrose, 2 KCl, 2 MgSO_4_, 1.25 NaH_2_PO_4_, 26 NaHCO_3_, 1 CaCl_2_, 1 MgCl_2_, and 10 D-glucose. After slice recovery at room temperature, the slices were transferred to a submerged recording chamber and were perfused with ACSF (2–3 mL/min) containing (in mM) 124 NaCl, 2.5 KCl, 1.3 MgSO_4_, 1 NaH_2_PO_4_, 26.2 NaHCO_3_, 2.5 CaCl_2_, 1.6 MgCl_2_, and 11 D-glucose. The solution was saturated with 95% O_2_/5% CO_2_ (Osmolarity, 290 ± 5 mOsm). Whole-cell patch clamp recordings were obtained from visually identified CA1 pyramidal neurons with a glass electrode (4–5 MΩ, Hilgenberg, Germany) containing (in mM) 120 K-gluconate, 10 KCl, 3 MgCl_2_, 0.5 EGTA, 40 HEPES, 2 MgATP, and 0.3 NaGTP (pH 7.2 with KOH, 295 mOsm) for measuring neuronal excitability. In the current clamp configuration, cells were held at −70 mV and injected from −75 mV to +400 pA with 25 pA increments. For measuring miniature excitatory postsynaptic currents (mEPSCs), 5 mM QX314 was added into the intracellular pipette solution while GABA_A_ receptor antagonist picrotoxin (PTX, 50 µM, Tocris, Bristol, UK), GABA_B_ receptor antagonist CGP55845 (3 µM, Tocris, Bristol, UK), and Na^+^ channel blocker tetrodotoxin (TTX, 1 µM, Tocris, Bristol, UK) were added to ACSF.

Miniature inhibitory postsynaptic currents (mIPSCs) were recorded with a glass electrode containing (in mM) 120 CsCl, 8 NaCl, 0.2 MgCl_2_, 10 HEPES, 2 EGTA, 0.3 Na_3_GTP, and 2 MgATP at pH 7.2 with CsOH, 290 mOsm. NBQX (25 µM, Tocris, Bristol, UK), D-APV (50 µM, Tocris, Bristol, UK), and TTX (1 µM) were added to ACSF to isolate action potential-independent mIPSCs. In vitro electrophysiological data were acquired using an EPC 10 amplifier (HEKA Elektronik, Germany) at a sampling rate of 10 kHz and low-pass-filtered at 2–3 kHz. The obtained data were analyzed offline using PatchMaster software v2X69 (HEKA Electronik, Germany), Clampfit 10 (Molecular Devices, U.S.A.), or MiniAnalysis (6.0.3 Synaptosoft, U.S.A.). The data were presented and analyzed using SigmaPlot 12 (Systat Software Inc, U.S.A.) and Prism 7 (GraphPad software, U.S.A.).

### 2.3. Immunocytochemistry in Hippocampal Cultured Neurons

Hippocampal neurons from embryonic C57BL6/J mice (E18) were extracted and cultured as described earlier [19]. The neuronal cells were plated on polyethyleneimine-coated (Sigma-Aldrich; 408727-100 mL) 18 mm coverslips in 12-well plates at a cell density of 150,000 per well. Neurons were maintained in 1 mL of neurobasal media (NB+ media) (Thermo Fisher Scientific, Waltham, MA, USA) containing 2% B27 and 1% L-glutamine and 1% Pen-Strep (Gibco, Grand Island, NY, USA). Cultured neurons were fed with 250 µL of NB+ media on days in vitro (DIV) 14 and 17. On DIV 21–23, cultured hippocampal neurons were incubated with Heparinase-1 (0.5 U/mL, Sigma-Aldrich, H2519, MO, USA), Ctrl (heat-inactivated Heparinase-1), or Heparinase-1 + AIP (100 nM), as previously described [19], for 2 h at 37 °C. After the treatment, hippocampal neurons were washed with phosphate-buffered saline (PBS) and fixed with 4% paraformaldehyde (PFA) for 10 min, and then permeabilized with 0.1% Triton-X-100 in PBS for 10 min, washed 3 times, and blocked (0.1% Glycine + 0.1% Tween-20 + 10% Normal Goat Serum in PBS) for 60 min at room temperature. Then, the cells were stained with antibodies against AnkG (mouse monoclonal, 1:1000; Millipore, MABN466), pCaMKII (rabbit polyclonal, 1:1000; Phospho Solution, P-1005-286), MAP2 (chicken polyclonal, 1:500; Abcam, ab5543) and DAPI (Life Technologies, S36939), and finally mounted (Fluoromount; Sigma Aldrich, F4680-25ML, MO, USA) for imaging. Mounted coverslips were imaged using a Zeiss LSM 700 confocal microscope with a 63×/1.4 NA oil immersion objective. Image analysis was carried out as previously described [19]. Using the microtubule-associated protein 2 (MAP2) and AnkG signals, the AISs were analyzed from the soma edge over a 40 µm long distance with a line profile (width = 3.0) using Fiji (ImageJ version 1.53c) [19].

### 2.4. In Vivo Intrahippocampal Injection

Adult (2- to 4-month-old) male C57Bl/6j mice (Charles River) were used. At least 1 week before starting the experiments, mice were transferred to a small vivarium, where they were housed individually with food and water ad libitum on a reversed 12:12 light/dark cycle (light on at 9:00 p.m). All behavioral experiments were performed in the afternoons during the dark phase of the cycle when the mice were active, under constant temperature (22 ± 1 °C) and humidity (55 ± 5%). All treatments and behavioral procedures were conducted in accordance with ethical animal research standards defined by German law and approved by the Ethical Committee on Animal Health and Care of the State of Saxony-Anhalt, Germany, under the license numbers 42502-2-1159 and -1322 DZNE.

Injection guide cannulas and electrodes were implanted as previously described [19], but electrophysiological analysis is not included in the present study due to an insufficient quality of recordings. The coordinates for bilateral cannulas were AP = 2.0 mm and L = ±2.2 mm from bregma and midline, respectively. For intrahippocampal injection, we used a digitally controlled infusion system (UltraMicroPump, UMP3, and Micro4 Controller, WPI, U.S.A.) fed with a 10 μL Hamilton syringe and a NanoFil (35 GA) bevel-tip needle, as previously described [19]. The mouse was first anesthetized with 1–3% isoflurane and put into the stereotaxic frame. Heparinase 1 (Hep) from *Flavobacterium heparinum* (0.05 U/µL/site, Sigma-Aldrich, H2519), Heparinase 1 heat-inactivated at 100 ºC for 30 min (Ctrl), or Heparinase 1 + autocamtide-2-related inhibitory peptide (0.17 µg/µL/site, Sigma-Aldrich, SCP0001) (Hep + AIP) was injected into the hippocampal CA1 area at a rate of 3 nl/s. After waiting for another 5 min, the injection needle was removed.

### 2.5. Fear Conditioning

In this study, we used the previously described classical Pavlovian contextual fear conditioning paradigm in mice with a slight modification [19]. In this study, on day 0 (d0), mice were initially placed in a 20 × 20 × 30 cm chamber with a neutral context (CC-), gray walls, and gray plastic floor for 5 min. Next, mice were exposed to the conditioned context (CC+), which includes patterned walls and a metal grid floor, for 5 min after an interval of 1 h. During the CC+ phase, mice’s feet were shocked 3 times with mild intensity (0.5 mA, 1 s) with a 1 min inter-shock interval. Using a computerized fear conditioning system (Ugo Basile, Italy), the first memory retrieval session was carried out for 5 min for each mouse on day 1 (d1) with a 1 h interval following the sequence CC- and CC+. On day 2 (d2), mice were injected with the vehicle, Heparinase and Heparinase + AIP, into the hippocampus. Then, on day 3 (d3) the second memory retrieval test (test 2) was performed using a similar paradigm as that used on d1. A blinded trained observer used video recordings of each session for offline fear-conditioned behavioral analysis with the help of behavioral video acquisition and analysis software (ANY-maze, version 4.99, Stoelting Co., Wood Dale, IL). Finally, the overall context memory and discrimination performance for each mouse was estimated.

### 2.6. Statistics

Numerical data are reported as mean ± SEM, with n being the number of samples. Student’s t-test and multi-way ANOVA with suitable post hoc tests were used as indicated and performed in SigmaPlot or Prism. For non-Gaussian distributions, we used the Mann–Whitney–Wilcoxon test. Significance levels (*p*-values) are indicated in figures by asterisks.

## 3. Results

### 3.1. Heparinase Treatment Elevates CaMKIIβ Autophosphorylation in the Mouse Hippocampus

**Figure 1:**
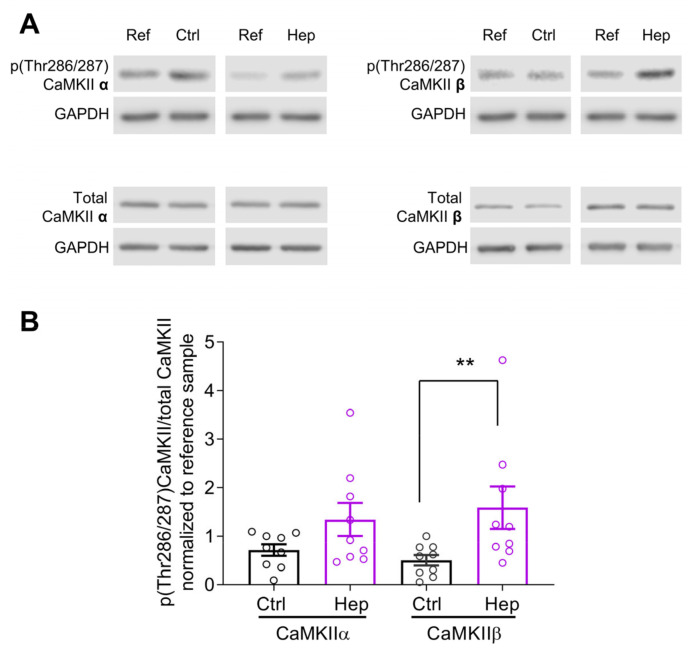
Heparinase treatment increased CaMKII activity in vivo. (**A**) Heparinase-injected mouse hippocampi were lysed, and extracts were used for immunoblotting. Membranes were incubated with anti-phospho-Thr286/287 CaMKII antibody to measure autophosphorylated CaMKII α and β levels relative to the total protein expression levels. (**B**) Summarized graph showing the statistical evaluation of Western blotting experiments, as in (**A**). Data are presented as means ± SEMs. Note that there was a significant increase in CaMKIIβ autophosphorylation one day after heparinase injection (** *p* < 0.01, Mann–Whitney–Wilcoxon test; Ctrl, *n* = 9; Hep, *n* = 9).

We previously observed an increase in the GluA1 expression and CaMKII activity in cultured mouse hippocampal neurons after heparinase 1 treatment [17]. To investigate whether heparinase treatment also changes hippocampal CaMKII activity in vivo, Ctrl (heat-inactivated heparinase) or active heparinase 1 was injected into the dorsal hippocampus of 6-week-old mice. To investigate the level of endogenous CaMKII isoform protein expression and their activity, 24 h after injection of heparinase, hippocampal slices were acutely prepared and used for immunoblotting. CaMKIIα and CaMKIIβ are major isoforms in the hippocampus, and these molecules are activated during memory formation. Activation of CaMKIIα and CaMKIIβ was assessed via the analysis of phosphorylation at Thr286 and Thr287, respectively [20]. Consistent with our previous observation for the cultured hippocampal neurons [17], the activity of CaMKIIβ was strongly affected after heparinase injection in vivo. The ratio of phosphorylated to total CaMKIIβ was increased after heparinase treatment, while the effect on CaMKIIα was less prominent (Figure 1).

### 3.2. Enzymatic Digestion of HSHSs Does Not Change Synaptic Transmission to CA1 Pyramidal Neurons

**Figure 2:**
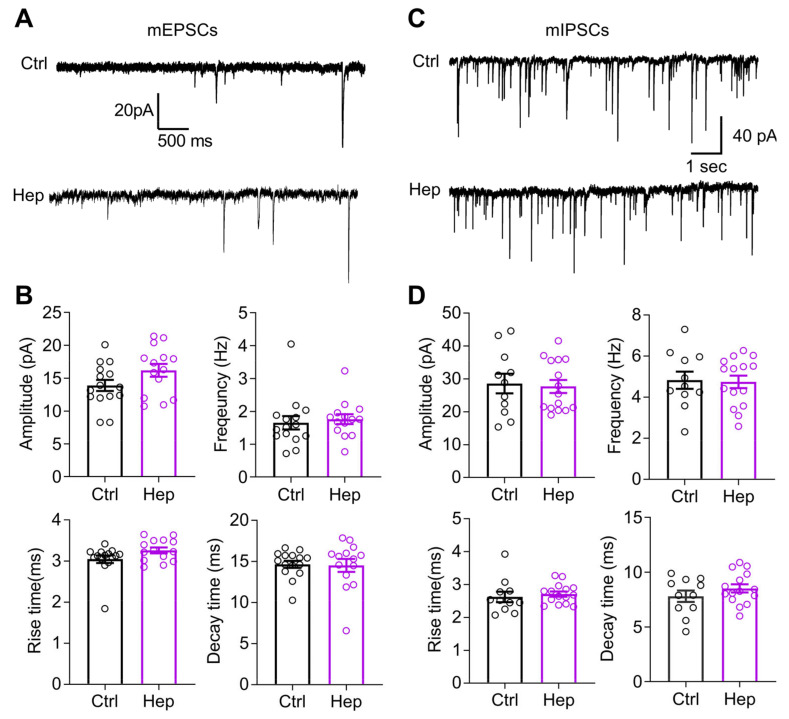
Intact excitatory and inhibitory synaptic transmission onto CA1 pyramidal cells one day after heparinase (Hep) injection into the mouse hippocampal CA1 in vivo. (**A**) Representative traces of mEPSCs from CA1 pyramidal cells in heat-inactivated (Ctrl) and active heparinase 1-injected mice in the presence of PTX, CGP55845, and TTX. (**B**) Summarized bar graphs show that amplitude, frequency, rise time, and decay time of mEPSCs remained intact after heparinase injection (*p* > 0.05, two-tailed *t*-test; Ctrl, *n* = 15; Hep, *n* = 14). Data are presented as means ± SEMs. (**C**) Representative traces of mIPSCs recorded from CA1 pyramidal cells in heat-inactivated (Ctrl) and active heparinase 1-injected mice in the presence of NBQX (25 µM), D-AP5 (50 µM), CGP55845 (3 µM), and TTX (1 µM). (**D**) Summarized bar graphs show that amplitude, frequency, rise time, and decay time of mIPSCs remained intact after heparinase injection into the hippocampal CA1 area (*p* > 0.05, two-tailed *t*-test; Ctrl, *n* = 15; Hep, *n* = 14). Data are presented as means ± SEMs.

Having found that heparinase treatment in vitro can up-scale mEPSCs, we measured glutamatergic transmission (mEPSC) and GABAergic transmission (mIPSC) to CA1 pyramidal neurons 1 day after heparinase injection in vivo. Unexpectedly, we did not observe changes in the mEPSCs’ amplitude or frequency (Figure 2A,C). Additionally, temporal parameters such as the rise and decay times were not affected (Figure 2A,C). The properties of mIPSCs were also unchanged by heparinase treatment (Figure 2B,D).

### 3.3. Impaired Neuronal Excitability after In Vivo Injection of Heparinase Is Rescued by CaMKII Inhibitor AIP

**Figure 3:**
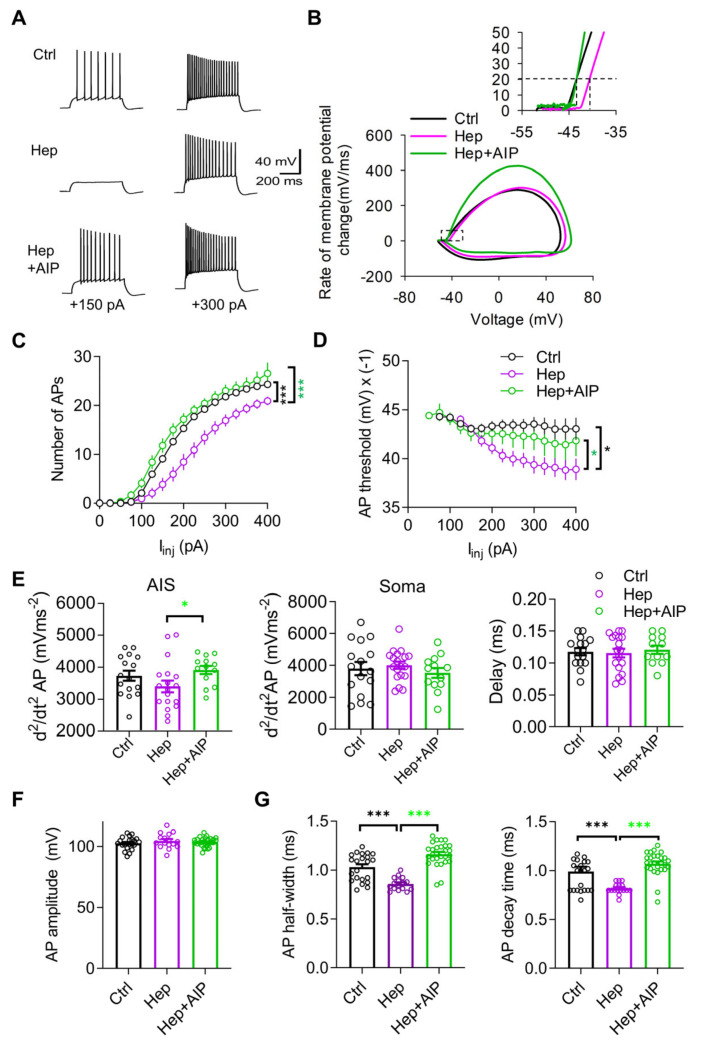
CaMKII inhibitor (AIP) could rescue reduced neuronal excitability of CA1 pyramidal neurons after heparinase (Hep) injection in vivo. (**A**) Sample traces of action potentials (APs) elicited by depolarizing current injections in the CA1 pyramidal cells. (**B**) Phase plot (dV/dt versus V) of the first action potential in response to 300 pA current injection. Inset (left) shows the initial phase of the action potential from the phase plot (right). (**C**,**D**) Summary graphs showing the number of action potentials elicited by different depolarizing current injections and the AP threshold at 20 mV/ms in response to different current injections. * *p* < 0.05, *** *p* < 0.001, Holm–Sidak test after two-way RM ANOVA. (**E**) Analysis of the amplitude and interval (Delay) between two peaks of the second action potential derivative (d^2^/dt^2^ AP), which correspond to the activation of sodium channels in the AIS and soma, revealed facilitated AP generation at the AIS after heparinase treatment with co-administration of AIP. * *p* < 0.05, Holm–Sidak test after one-way ANOVA. (**F**,**G**) Summary graphs comparing Ctrl (heat-inactivated heparinase), heparinase, and heparinase+AIP-injected neurons in terms of the amplitude (**F**), half-width (**G**), and decay time (**G**) of the action potential (Ctrl, *n* = 22; Hep, *n* = 17; Hep+AIP, *n* = 10; *** *p* < 0.05, Holm–Sidak test after one-way ANOVA). Data are presented as means ± SEMs.

Next, we investigated the excitability of CA1 pyramidal neurons. We previously reported that acute heparinase treatment of hippocampal slices reduced action potential (AP) probability during theta-burst stimulation and hence decreased Ca^2+^ influx to dendritic spines during the induction of LTP [19]. Based on that study, we expected that one day of heparinase treatment may also result in reduced neuronal excitability in the CA1 pyramidal cells. Therefore, we performed patch clamp recordings in the current clamp configuration and measured the number of APs as a function of injected currents (Figure 3A), the threshold of action potential generation (Figure 3B), and other parameters characterizing the magnitude and shape of APs (Figure 3C–F). To verify the role of CaMKII in shaping the effects of heparinase, we employed AIP as a selective and potent inhibitor of CaMKII, which has been used in slices and in vivo [21,22,23]. We co-injected AIP with heparinase one day before recordings.

Compared with the control group, fewer APs were evoked in response to depolarizing currents after injection of heparinase (Figure 3A). Analysis of input–output curves showing the average number of APs for each intensity of stimulation revealed a significant reduction in cell excitability in the heparinase-treated neurons and restoration of excitability by AIP (Figure 3C). Another indicator of excitability is the spike threshold (Scott et al., 2014). After the heparinase treatment, neurons started to fire at more positive membrane potential in the heparinase group as compared to the control, and this effect was abrogated by AIP (Figure 3D). Analysis of two peaks in the second derivative of APs, which correspond to AP generation at the AIS and soma [24], revealed a tendency toward reduction in the magnitude of the first peak after heparinase treatment (but not the second peak or the interval between peaks), and a significant increase in the first peak after CaMKII inhibition, suggesting the modulation of AIS excitability (Figure 3E). An axonal site of heparinase action is also indirectly suggested by the absence of heparinase effects on the peak spike voltage (AP amplitude), which represents an indicator of somatic sodium channel availability (Figure 3F). Heparinase also reduced, in a CaMKII-dependent manner, the half-width and decay of the action potentials, suggesting some modulation of potassium channels (Figure 3G).

### 3.4. Increased Activity of CaMKII and Impaired Expression of AnkG at the Axon Initial Segment after Heparinase Treatment Are Abrogated by AIP

**Figure 4:**
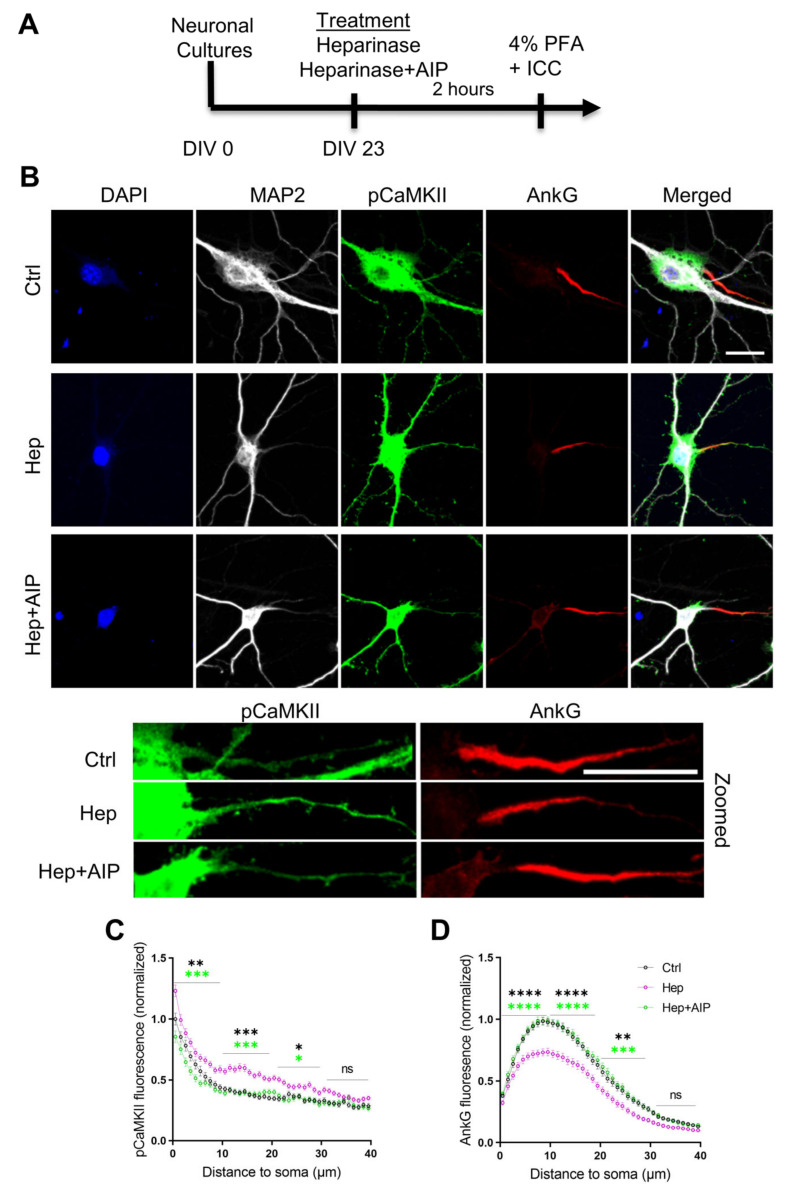
The CaMKII inhibitor AIP abrogates heparinase (Hep)-induced reduction in AnkG expression in the AIS of hippocampal neurons. (**A**) Timeline showing the treatment of hippocampal neurons with heparinase and AIP for 2 h followed by immunocytochemistry (ICC). (**B**) Hippocampal neurons were stained with DAPI (blue), MAP2 (gray), pCaMKII (green), and Ankyrin G (AnkG, red) antibodies. Scale bars for both original and zoomed images are 20 µm. (**C**,**D**) The pCaMKII (**C**) and AnkG (**D**) distributions at the AIS were determined by computing a 40 µm long fluorescence intensity profile with a thickness of 3 pixels in the middle of AnkG-immunopositive areas starting from the edge of the soma (determined using the MAP2 signal). The average expression of AnkG and pCaMKII along the 40 µm line profile of the AIS was quantified from 165 (Ctrl), 183 (Hep), and 167 (Hep+AIP) neurons from three independent experiments. Line profiles were normalized to the peak AnkG and pCaMKII values from the control group for each independent experiment. * *p* < 0.05, ** *p* < 0.01, *** *p* < 0.001, **** *p* < 0.0001, Holm–Sidak post hoc test after two-way RM ANOVA for the line profiles binned with 10 µm interval.

Our previous study revealed that the removal of HSHSs reduces AnkG expression at the AIS in vitro and in vivo [19]. As we in the present study observed the increased autophosphorylation of CaMKII after heparinase treatment, we investigated if the reduction in AnkG at the AIS correlates with changes in CaMKII phosphorylation at the same subcellular domain and whether the pharmacological inhibition of the CaMKII autophosphorylation with AIP could abrogate the effects of heparinase treatment on AnkG expression. To facilitate the quantitative analysis of protein expression in the AIS, it was performed in vitro as previously described [19]. We observed an increased level of pCaMKII at the AIS after digesting HSHSs, which was reduced by AIP to the control levels (Figure 4). Similar to our previous findings, the removal of HSHSs reduced the expression of AnkG along the 40 µm distance of the AIS relative to the control. In line with our electrophysiological recordings, co-incubating hippocampal neurons with heparinase and AIP restored the expression of AnkG to the control levels (Figure 4). Together with electrophysiological data, these results suggest that the reduction in AnkG expression at the AIS and reduced neuronal excitability after cleaving HSHSs are induced by the increased autophosphorylation of CaMKII.

### 3.5. Impaired Recall of Contextual Memories after Heparinase Treatment Is Rescued by Co-Administration of AIP

**Figure 5:**
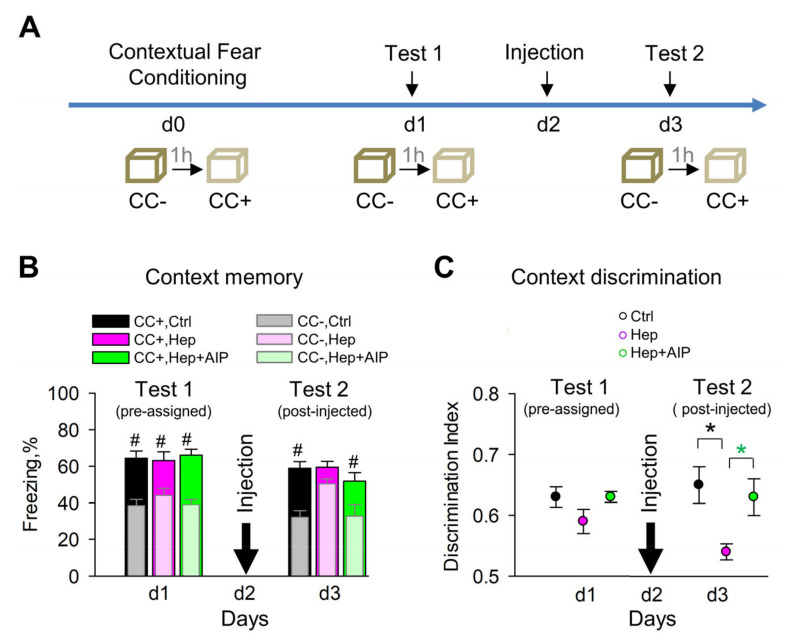
CaMKII inhibitor AIP rescues impaired context discrimination after heparinase (Hep) injection in vivo. (**A**) Scheme of the experiment. (**B**,**C**) Intrahippocampal heparinase injection 24 h after fear conditioning increases freezing in the neutral (CC-) context to the level measured in the conditioned (CC+) context (**B**) and impairs context discrimination (**C**), which can be rescued by inhibition of CaMKII with AIP. # *p* < 0.05, paired t-test to compare freezing in conditioned and neutral contexts. * *p* < 0.05, Holm–Sidak post hoc test after two-way RM ANOVA to compare treatments.

In our previous study, we found that heparinase injected before contextual fear conditioning did not affect the level of spontaneous freezing/immobility before conditioning but impaired context discrimination 24 h after conditioning [19]. This experiment, however, did not allow us to dissect whether HSHSs are essential for the acquisition, consolidation, or recall of contextual memories because re-expression of glycans is a slow process taking several weeks [25], and hence the removal of HSHSs before conditioning would result in impaired HSHS expression during acquisition, consolidation, and recall of memories for the next few days after conditioning. In the present study, we specifically tested if HSHSs are necessary for proper contextual memory recall by injecting heparinase on day 2 after contextual fear conditioning (Figure 5a), i.e., after the acquisition and consolidation of memories were successfully completed. This was confirmed by normal freezing time in the conditioned context and normal context discrimination on day 1 in mice pre-assigned to all experimental groups, i.e., control, heparinase, and heparinase plus AIP (Figure 5A). Additionally, on day 3 after conditioning, i.e., 24 h after heparinase injection, the freezing time in the conditioned context was normal in the control group, but heparinase-treated mice showed increased freezing in the neutral context CC- (Figure 5B) and impaired contextual discrimination (Figure 5C). Co-administration of AIP restored normal context discrimination after heparinase treatment, not affecting freezing time in the conditioned context.

## 4. Discussion

Our data show that enzymatic removal of HSHSs in the CA1 region of the hippocampus does not affect miniature postsynaptic currents but leads to reduced axonal excitability of pyramidal cells and impaired contextual discrimination, which correlate with increased activity of CaMKII in general, but particularly in the AIS. Inhibition of CaMKII with AIP normalizes excitability and expression of AnkG in the AIS after heparinase treatment, suggesting a causal link between HSPGs and regulation of axonal excitability via CaMKII autophosphorylation. Below, we discuss the functional importance and possible molecular mechanisms underlying these findings.

Highly expressed in excitatory synapses in the hippocampus, CaMKII has been studied in many aspects of synaptic function, such as synaptic strength and synaptic plasticity. Overexpression of α and β isoforms of CaMKII in cultured neurons has the opposite effects on mEPSCs’ strength frequency while CaMKIIβ-overexpressing cells exhibit an increase [26]. Thus, it is plausible to assume that in our experiments, the effects of increased CaMKIIβ activation were counterbalanced by increased activity of synaptic CaMKIIα, but we cannot exclude the saturation of CaMKIIβ effects under in vivo conditions of the present experiments.

The autophosphorylation of CaMKII, especially that of CaMKIIα, on the other hand, has been shown to reduce the excitability of CA1 neurons, which may impact learning [27,28], while inhibition of CaMKIIα autophosphorylation by a point mutation at T286A increased CA1 neuron excitability. These data are in line with our finding that autophosphorylation of CaMKII was increased after heparinase treatment in the AIS, while expression of AnkG was impaired but could be rescued by the AIP co-administration. Studies show that AIP specifically inhibits CaMKII relative to other kinases, such as protein kinase C (PKC), CAMKI, and CaMKIV, in rat brain extracts [29,30] and in mice [22,23]. The degree of specificity of AIP effects on CaMKIIα versus CaMKIIβ, however, has not been properly resolved.

The AIS, located between axonal and somatodendritic domains, is a key structure for the initiation of action potential firing. AnkG, neuronal cell adhesion molecule (NrCAM), βIV-spectrin, and voltage-gated sodium and potassium channels are major structural/functional components of the AIS, and their alteration affects AIS assembly and function [31]. βIV-spectrin, an AnkG interaction partner, serves as a bridge between AnkG and the actin-based cytoskeleton. Accordingly, animal models harboring AnkG gene deficiency exhibit abnormal animal behavior (such as ataxia) and neuronal excitability in the cerebellum, due to the mislocalization of sodium channels [32]. Progressive ataxia and tremors are also observed in different βIV-spectrin mutant mice (qv^3J^ and βIV-null mice) [33]. Findings of the mislocalization of sodium channels in the AIS of cerebellar and hippocampal neurons in these mutant mice suggest that altered sodium channel expression is responsible for the neurological phenotypes of the mutants. In the cardiomyocyte, βIV-spectrin’s interaction with CaMKII leads to sodium channel phosphorylation via βIV-dependent targeting of CaMKII [34]. The abnormal kinetics of sodium channels and altered cellular excitability after a loss-of-function mutation in the βIV-spectrin gene in the qv^3J^ mouse line suggest that the βIV-spectrin/CaMKII complex is an important component for Na^+^ channel regulation in cardiomyocytes. Interestingly, CaMKII is colocalized with βIV spectrin in the AIS of cerebellar Purkinje neurons as well, and qv^3J^ mutant mice exhibit a relatively weak immunostaining signal of CaMKII in the AIS of Purkinje cells, implying that the βIV-spectrin/CaMKII complex would strongly affect cellular excitability in both the heart and the brain [34]. Thus, further studies are warranted to study the distribution of βIV-spectrin and ion channels in the AIS after the targeting of HSHSs.

Extracellularly, the secreted protein gliomedin is a key component at the nodes of Ranvier in the peripheral nerves. The deposition of gliomedin multimers at the nodal gaps facilitates the clustering of the axonodal cell adhesion molecules neurofascin and NrCAM and sodium channels by binding to HSPGs [35]. In cortical neurons, agrin binds to a tyrosine kinase receptor, which results in the elevation of intracellular Ca^2+^ and subsequent activation of the CaMKII signaling pathway [36]. Regarding potential protein carriers of HSHSs responsible for the regulation of CaMKII activity at the AIS, there are several candidates. Glypicans 1 and 2 are expressed axonally [37,38]. Glypican-4 is also enriched on hippocampal granule cell axons and can bind to its partner orphan receptor, GPR158 [39]. Additionally, syndecans are known to be localized at the nodes of Ranvier [40] and axons [3,41]. Syndecans 2 and 3 can directly bind to CASK protein via the PDZ domain [4] that regulates CaMKII activity in neurons [42]. Further studies on the AIS in mice deficient in these HSPGs could be instrumental to identify their role in AIS assembly and axonal excitability via regulation of CaMKII.

Our behavioral experiments for the first time suggest the role of HSHSs in the proper recall of contextual memories and show that in vivo inhibition of CaMKII by AIP could abrogate the hypergeneralization induced by heparinase. Previously, AIP has been shown to significantly protect neurons from NMDA-induced neurotoxicity [43], fully restore contractility in cardiac muscles of diabetic rats [44], inhibit doxorubicin-induced apoptosis of cardiac cells [45], and prevent the reinstatement of morphine-seeking behavior in rats [46]. As hypergeneralization is common for several conditions [47,48,49], targeting this mechanism might be of therapeutic value.

A similar loss of context discrimination is found when contextual memories are transferred from the hippocampus to the anterior cingulate cortex via the retrosplenial cortex. Moreover, high-frequency stimulation of memory engrams in the retrosplenial cortex one day after learning produces a recent memory with features normally observed in consolidated remote memories, including contextual generalization and decreased hippocampal dependence [50]. Thus, our data are consistent with the hypothesis that the recent contextual memory is distributed in several brain areas and, if the hippocampal engrams, in particular CA1, are not activated enough due to a loss of excitability induced by heparinase, another, presumably cortical, representation is used.

In summary, our data make a stronger link between HSHSs and regulation of neuronal excitability and implicate CaMKII in this regulation. Aberrant expression or activity of HSPGs is associated with some pathological conditions, such as glioblastoma, Fragile X syndrome, neuroinflammation, and Parkinson’s disease [51,52,53,54]. Additionally, HSPGs are known to bind and co-aggregate with amyloid beta (Aβ) [55,56]. In light of reported neuronal hyperexcitability in Alzheimer’s patients and models of Alzheimer’s disease [57,58], our work suggests that Aβ-HSPG interactions may affect the expression of HSPGs at the AIS, decreasing activation of CaMKII at the AIS and hence increasing neuronal excitability. At synapses, Aβ is known to inhibit autophosphorylation of CaMKII at Thr286 and impair synaptic plasticity [59]. Thus, our study suggests potential pathophysiological mechanisms and indicates an option to prevent these by targeting CaMKII signaling at the AIS.

## Data Availability

Data supporting reported results can be obtained upon a request to the corresponding author (A.D.).

## References

[1] Ford-Perriss M., Turner K., Guimond S., Apedaile A., Haubeck H.D., Turnbull J., Murphy M. (2003). Localisation of specific heparan sulfate proteoglycans during the proliferative phase of brain development. Dev. Dyn..

[2] Farhy-Tselnicker I., van Casteren A.C.M., Lee A., Chang V.T., Aricescu A.R., Allen N.J. (2017). Astrocyte-Secreted Glypican 4 Regulates Release of Neuronal Pentraxin 1 from Axons to Induce Functional Synapse Formation. Neuron.

[3] Hsueh Y.P., Sheng M. (1999). Regulated expression and subcellular localization of syndecan heparan sulfate proteoglycans and the syndecan-binding protein CASK/LIN-2 during rat brain development. J. Neurosci..

[4] Hsueh Y.P., Yang F.C., Kharazia V., Naisbitt S., Cohen A.R., Weinberg R.J., Sheng M. (1998). Direct interaction of CASK/LIN-2 and syndecan heparan sulfate proteoglycan and their overlapping distribution in neuronal synapses. J. Cell Biol..

[5] Grootjans J.J., Zimmermann P., Reekmans G., Smets A., Degeest G., Durr J., David G. (1997). Syntenin, a PDZ protein that binds syndecan cytoplasmic domains. Proc. Natl. Acad. Sci. USA.

[6] Gao Y., Li M., Chen W., Simons M. (2000). Synectin, syndecan-4 cytoplasmic domain binding PDZ protein, inhibits cell migration. J. Cell Physiol..

[7] Ethell I.M., Hagihara K., Miura Y., Irie F., Yamaguchi Y. (2000). Synbindin, A novel syndecan-2-binding protein in neuronal dendritic spines. J. Cell Biol..

[8] Ethell I.M., Yamaguchi Y. (1999). Cell surface heparan sulfate proteoglycan syndecan-2 induces the maturation of dendritic spines in rat hippocampal neurons. J. Cell Biol..

[9] Zimmermann P., Zhang Z., Degeest G., Mortier E., Leenaerts I., Coomans C., Schulz J., N’Kuli F., Courtoy P.J., David G. (2005). Syndecan recycling [corrected] is controlled by syntenin-PIP2 interaction and Arf6. Dev. Cell.

[10] Lauri S.E., Kaukinen S., Kinnunen T., Ylinen A., Imai S., Kaila K., Taira T., Rauvala H. (1999). Regulatory role and molecular interactions of a cell-surface heparan sulfate proteoglycan (N-syndecan) in hippocampal long-term potentiation. J. Neurosci..

[11] Kaksonen M., Pavlov I., Voikar V., Lauri S.E., Hienola A., Riekki R., Lakso M., Taira T., Rauvala H. (2002). Syndecan-3-deficient mice exhibit enhanced LTP and impaired hippocampus-dependent memory. Mol. Cell. Neurosci..

[12] McCroskery S., Bailey A., Lin L., Daniels M.P. (2009). Transmembrane agrin regulates dendritic filopodia and synapse formation in mature hippocampal neuron cultures. Neuroscience.

[13] Coles C.H., Shen Y., Tenney A.P., Siebold C., Sutton G.C., Lu W., Gallagher J.T., Jones E.Y., Flanagan J.G., Aricescu A.R. (2011). Proteoglycan-specific molecular switch for RPTPsigma clustering and neuronal extension. Science.

[14] Dityatev A., Dityateva G., Sytnyk V., Delling M., Toni N., Nikonenko I., Muller D., Schachner M. (2004). Polysialylated neural cell adhesion molecule promotes remodeling and formation of hippocampal synapses. J. Neurosci..

[15] Irie F., Badie-Mahdavi H., Yamaguchi Y. (2012). Autism-like socio-communicative deficits and stereotypies in mice lacking heparan sulfate. Proc. Natl. Acad. Sci. USA.

[16] Zhang P., Lu H., Peixoto R.T., Pines M.K., Ge Y., Oku S., Siddiqui T.J., Xie Y., Wu W., Archer-Hartmann S. (2018). Heparan Sulfate Organizes Neuronal Synapses through Neurexin Partnerships. Cell.

[17] Korotchenko S., Cingolani L.A., Kuznetsova T., Bologna L.L., Chiappalone M., Dityatev A. (2014). Modulation of network activity and induction of homeostatic synaptic plasticity by enzymatic removal of heparan sulfates. Philos. Trans. R. Soc. London Ser. B Biol. Sci..

[18] Garau G., Magotti P., Heine M., Korotchenko S., Lievens P.M., Berezin V., Dityatev A. (2015). Heparin/heparan sulfates bind to and modulate neuronal L-type (Cav1.2) voltage-dependent Ca(2+) channels. Exp. Neurol..

[19] Minge D., Senkov O., Kaushik R., Herde M.K., Tikhobrazova O., Wulff A.B., Mironov A., van Kuppevelt T.H., Oosterhof A., Kochlamazashvili G. (2017). Heparan Sulfates Support Pyramidal Cell Excitability, Synaptic Plasticity, and Context Discrimination. Cereb. Cortex.

[20] Baucum A.J., Shonesy B.C., Rose K.L., Colbran R.J. (2015). Quantitative proteomics analysis of CaMKII phosphorylation and the CaMKII interactome in the mouse forebrain. ACS Chem. Neurosci..

[21] De Carvalho Myskiw J., Furini C.R., Benetti F., Izquierdo I. (2014). Hippocampal molecular mechanisms involved in the enhancement of fear extinction caused by exposure to novelty. Proc. Natl. Acad. Sci. USA.

[22] Johnson C.N., Pattanayek R., Potet F., Rebbeck R.T., Blackwell D.J., Nikolaienko R., Sequeira V., Le Meur R., Radwanski P.B., Davis J.P. (2019). The CaMKII inhibitor KN93-calmodulin interaction and implications for calmodulin tuning of NaV1.5 and RyR2 function. Cell Calcium.

[23] Murakoshi H., Shin M.E., Parra-Bueno P., Szatmari E.M., Shibata A.C.E., Yasuda R. (2017). Kinetics of Endogenous CaMKII Required for Synaptic Plasticity Revealed by Optogenetic Kinase Inhibitor. Neuron.

[24] Meeks J.P., Mennerick S. (2007). Action potential initiation and propagation in CA3 pyramidal axons. J. Neurophysiol..

[25] Romberg C., Yang S., Melani R., Andrews M.R., Horner A.E., Spillantini M.G., Bussey T.J., Fawcett J.W., Pizzorusso T., Saksida L.M. (2013). Depletion of perineuronal nets enhances recognition memory and long-term depression in the perirhinal cortex. J. Neurosci..

[26] Thiagarajan T.C., Piedras-Renteria E.S., Tsien R.W. (2002). alpha- and betaCaMKII. Inverse regulation by neuronal activity and opposing effects on synaptic strength. Neuron.

[27] Sametsky E.A., Disterhoft J.F., Ohno M. (2009). Autophosphorylation of alphaCaMKII downregulates excitability of CA1 pyramidal neurons following synaptic stimulation. Neurobiol. Learn. Mem..

[28] Giese K.P., Fedorov N.B., Filipkowski R.K., Silva A.J. (1998). Autophosphorylation at Thr286 of the alpha calcium-calmodulin kinase II in LTP and learning. Science.

[29] Pellicena P., Schulman H. (2014). CaMKII inhibitors: From research tools to therapeutic agents. Front. Pharmacol..

[30] Ishida A., Kameshita I., Okuno S., Kitani T., Fujisawa H. (1995). A novel highly specific and potent inhibitor of calmodulin-dependent protein kinase II. Biochem. Biophys. Res. Commun..

[31] Huang C.Y., Rasband M.N. (2018). Axon initial segments: Structure, function, and disease. Ann. N. Y. Acad. Sci..

[32] Zhou D., Lambert S., Malen P.L., Carpenter S., Boland L.M., Bennett V. (1998). AnkyrinG is required for clustering of voltage-gated Na channels at axon initial segments and for normal action potential firing. J. Cell Biol..

[33] Komada M., Soriano P. (2002). [Beta]IV-spectrin regulates sodium channel clustering through ankyrin-G at axon initial segments and nodes of Ranvier. J. Cell Biol..

[34] Hund T.J., Koval O.M., Li J., Wright P.J., Qian L., Snyder J.S., Gudmundsson H., Kline C.F., Davidson N.P., Cardona N. (2010). A beta(IV)-spectrin/CaMKII signaling complex is essential for membrane excitability in mice. J. Clin. Investig..

[35] Eshed Y., Feinberg K., Carey D.J., Peles E. (2007). Secreted gliomedin is a perinodal matrix component of peripheral nerves. J. Cell Biol..

[36] Hilgenberg L.G., Smith M.A. (2004). Agrin signaling in cortical neurons is mediated by a tyrosine kinase-dependent increase in intracellular Ca^2+^ that engages both CaMKII and MAPK signal pathways. J. Neurobiol..

[37] Ivins J.K., Litwack E.D., Kumbasar A., Stipp C.S., Lander A.D. (1997). Cerebroglycan, a developmentally regulated cell-surface heparan sulfate proteoglycan, is expressed on developing axons and growth cones. Dev. Biol..

[38] Litwack E.D., Ivins J.K., Kumbasar A., Paine-Saunders S., Stipp C.S., Lander A.D. (1998). Expression of the heparan sulfate proteoglycan glypican-1 in the developing rodent. Dev. Dyn..

[39] Condomitti G., Wierda K.D., Schroeder A., Rubio S.E., Vennekens K.M., Orlandi C., Martemyanov K.A., Gounko N.V., Savas J.N., de Wit J. (2018). An Input-Specific Orphan Receptor GPR158-HSPG Interaction Organizes Hippocampal Mossy Fiber-CA3 Synapses. Neuron.

[40] Goutebroze L., Carnaud M., Denisenko N., Boutterin M.C., Girault J.A. (2003). Syndecan-3 and syndecan-4 are enriched in Schwann cell perinodal processes. BMC Neurosci..

[41] Luo N., Li H., Xiang B., Qiao L., He J., Ji Y., Liu Y., Li S., Lu R., Li Y. (2016). Syndecan-4 modulates the proliferation of neural cells and the formation of CaP axons during zebrafish embryonic neurogenesis. Sci. Rep..

[42] Lu C.S., Hodge J.J., Mehren J., Sun X.X., Griffith L.C. (2003). Regulation of the Ca2+/CaM-responsive pool of CaMKII by scaffold-dependent autophosphorylation. Neuron.

[43] Laabich A., Cooper N.G. (2000). Neuroprotective effect of AIP on N-methyl-D-aspartate-induced cell death in retinal neurons. Brain Res. Mol. Brain Res..

[44] Daniels L.J., Wallace R.S., Nicholson O.M., Wilson G.A., McDonald F.J., Jones P.P., Baldi J.C., Lamberts R.R., Erickson J.R. (2018). Inhibition of calcium/calmodulin-dependent kinase II restores contraction and relaxation in isolated cardiac muscle from type 2 diabetic rats. Cardiovasc. Diabetol..

[45] Ikeda S., Matsushima S., Okabe K., Ikeda M., Ishikita A., Tadokoro T., Enzan N., Yamamoto T., Sada M., Deguchi H. (2019). Blockade of L-type Ca(2+) channel attenuates doxorubicin-induced cardiomyopathy via suppression of CaMKII-NF-kappaB pathway. Sci. Rep..

[46] Liu Z., Zhang J.J., Liu X.D., Yu L.C. (2012). Inhibition of CaMKII activity in the nucleus accumbens shell blocks the reinstatement of morphine-seeking behavior in rats. Neurosci. Lett..

[47] Duits P., Cath D.C., Lissek S., Hox J.J., Hamm A.O., Engelhard I.M., van den Hout M.A., Baas J.M. (2015). Updated meta-analysis of classical fear conditioning in the anxiety disorders. Depress. Anxiety.

[48] Vlaeyen J.W.S., Linton S.J. (2012). Fear-avoidance model of chronic musculoskeletal pain: 12 years on. Pain.

[49] Dymond S., Dunsmoor J.E., Vervliet B., Roche B., Hermans D. (2015). Fear Generalization in Humans: Systematic Review and Implications for Anxiety Disorder Research. Behav. Ther..

[50] De Sousa A.F., Cowansage K.K., Zutshi I., Cardozo L.M., Yoo E.J., Leutgeb S., Mayford M. (2019). Optogenetic reactivation of memory ensembles in the retrosplenial cortex induces systems consolidation. Proc. Natl. Acad. Sci. USA.

[51] Kazanskaya G.M., Tsidulko A.Y., Volkov A.M., Kiselev R.S., Suhovskih A.V., Kobozev V.V., Gaytan A.S., Aidagulova S.V., Krivoshapkin A.L., Grigorieva E.V. (2018). Heparan sulfate accumulation and perlecan/HSPG2 up-regulation in tumour tissue predict low relapse-free survival for patients with glioblastoma. Histochem. Cell Biol..

[52] Sears J.C., Broadie K. (2017). Fragile X Mental Retardation Protein Regulates Activity-Dependent Membrane Trafficking and Trans-Synaptic Signaling Mediating Synaptic Remodeling. Front. Mol. Neurosci..

[53] Bussini S., Meda L., Scarpini E., Clementi E., Conti G., Tiriticco M., Bresolin N., Baron P. (2005). Heparan sulfate proteoglycan induces the production of NO and TNF-alpha by murine microglia. Immun. Ageing.

[54] Chen M., Vincent J., Ezeanii A., Wakade S., Yerigenahally S., Mor D.E. (2022). Heparan sulfate proteoglycans mediate prion-like alpha-synuclein toxicity in Parkinson’s in vivo models. Life Sci. Alliance.

[55] Liu C.C., Zhao N., Yamaguchi Y., Cirrito J.R., Kanekiyo T., Holtzman D.M., Bu G. (2016). Neuronal heparan sulfates promote amyloid pathology by modulating brain amyloid-beta clearance and aggregation in Alzheimer’s disease. Sci. Transl. Med..

[56] Van Horssen J., Wesseling P., van den Heuvel L.P., de Waal R.M., Verbeek M.M. (2003). Heparan sulphate proteoglycans in Alzheimer’s disease and amyloid-related disorders. Lancet Neurol..

[57] Kazim S.F., Seo J.H., Bianchi R., Larson C.S., Sharma A., Wong R.K.S., Gorbachev K.Y., Pereira A.C. (2021). Neuronal Network Excitability in Alzheimer’s Disease: The Puzzle of Similar versus Divergent Roles of Amyloid beta and Tau. eNeuro.

[58] Lam A.D., Deck G., Goldman A., Eskandar E.N., Noebels J., Cole A.J. (2017). Silent hippocampal seizures and spikes identified by foramen ovale electrodes in Alzheimer’s disease. Nat. Med..

[59] Zhao D., Watson J.B., Xie C.W. (2004). Amyloid beta prevents activation of calcium/calmodulin-dependent protein kinase II and AMPA receptor phosphorylation during hippocampal long-term potentiation. J. Neurophysiol..

